# The Legacy of the Great Hunger: Obesity, Hypothalamic Circuits, and the Role of Tryptophan: A Proposed Mechanism

**DOI:** 10.3390/brainsci16101035

**Published:** 2026-09-27

**Authors:** Malak Albedawe, Julian Paul Keenan

**Affiliations:** Cognitive Neuroimaging Laboratory, Montclair State University, 320 Reid Hall, Montclair, NJ 07043, USA

**Keywords:** obesity, brain, tryptophan, serotonin, famine, hypothalamus, the great hunger

## Abstract

**Highlights:**

**What are the main findings?**

- The Great Hunger, also known as the Irish Potato Famine of 1845–1852, is proposed to have substantially reduced tryptophan levels, which may have contributed to increased rates of neurological, physical and mental health issues.
- The effects may have been transgenerational, and here we propose how that could be possible via hypothalamic mechanisms.

**What are the implications of the main findings?**

- Famines and population food deprivations impact a wide range of physical systems including the brain.
- The Irish Great Hunger may have epigenetic effects, which are controversial, but here we propose potential mechanisms.

**Abstract:**

Ireland has elevated rates of obesity; Eurostat EU-SILC data indicate Ireland ranks approximately 12th in the EU for combined overweight and obesity among adults, and the usual explanations including diet, inactivity, and socioeconomic conditions do not fully account for the burden of metabolic disease. Here we argue that part of the answer may originate in The Great Hunger (An Gorta Mór) which took place in Ireland from 1845 to 1852. The blighting of the potato crop by *Phytophthora infestans* and seven years of malnutrition left most of the rural Irish population without their only meaningful source of tryptophan and vitamin B6, the two nutrients the body needs to synthesize serotonin. Fetuses developing during those years may therefore have formed hypothalamic appetite circuits under reduced serotonin availability, potentially resulting in reduced expression of the receptor that signals satiety in the brain. Evidence from the Dutch Hunger Winter, the Great Chinese Famine, Finland, Ghana, and the Holodomor indicates that this type of prenatal nutritional disruption is consistently associated with elevated obesity and metabolic disease risk in adulthood, and that some of the associated epigenetic differences are detectable in subsequent generations, although germline transmission has not been established in humans. Here we explore potential neural epigenetic mechanisms that may have originated in the Great Hunger and in subsequent generations.

## 1. Introduction

Ireland records elevated rates of overweight and obesity; approximately 61% of adults are classified as overweight or obese [1,2]. Obesity in Ireland is associated with elevated rates of type 2 diabetes, cardiovascular disease, hypertension, metabolic syndrome, and increased cancer risk [2,3]. Donovan and McNulty [2] document a three-decade trajectory of rising Irish obesity prevalence, from 8% of men and 13% of women in 1990 to substantially higher rates by 2010, and that this epidemiological burden is not adequately accounted for by contemporary diet quality, physical activity, or socioeconomic variables, which are comparable to northern European nations. The present review argues that a component of this excess metabolic load reflects potential neurobiological contributions of the Great Hunger of 1845 to 1852, working through epigenetic programming of hypothalamic serotonin circuits during a critical prenatal developmental window.

Before The Great Hunger, the Irish rural poor were not a nutritionally compromised population. Starting around 1815, Irish recruits in the British Army stood roughly 1–2 cm taller than English soldiers, a difference attributed to the relatively beneficial nutrition that a diet of almost exclusively potatoes and buttermilk provided [4,5]. The British urban working class experienced a measurable decline in height initially from the 1820s, explained by a decline in nutrition and welfare status and an increase in social inequality [6]. Skeletal analysis of the Kilkenny Union Workhouse mass burials from The Great Hunger time period confirms this baseline directly. Stature estimates for the Kilkenny population fell within the normal range for 19th century Irish, British, and American skeletal populations, and were in fact higher than those recorded for the Christ Church Spitalfields population in London. Geber [7] concludes that the Kilkenny workhouse inmates did not exhibit shorter stature because of their social and economic background. The population that *entered* The Great Hunger was therefore not nutritionally depleted. The blighting of the potato was a deprivation imposed on a healthy population, which further supports the biological significance of the epigenetic changes that followed.

The blight resulted from the repeated failure of the potato crop caused by the oomycete *Phytophthora infestans* and reduced the island’s population by approximately 25% through mortality and emigration [4,5,6]. As indicated, the potato formed the majority of dietary protein and micronutrient intake, including all dietary tryptophan and pyridoxine (Vitamin B6), the cofactor required for tryptophan hydroxylase activity in serotonin synthesis [8]. The long-term collapse of the potato therefore produced a specific, sustained deprivation of the substrate, which central and peripheral serotonin synthesis depends on.

Tryptophan cannot be endogenously synthesized and must be supplied through diet. During fetal development, serotonin functions not only as a neurotransmitter but as a trophic morphogen regulating the proliferation, migration, and differentiation of neurons forming the hypothalamic feeding circuit [9]. The satiety mediating 5-HT2C receptor (5-HT2CR), expressed on arcuate nucleus pro-opiomelanocortin (POMC) neurons, is established during a defined perinatal window sensitive to maternal amino acid availability [10,11]. Maternal protein and tryptophan restriction reduces fetal hypothalamic 5-HT2CR expression and produces constitutive hyperphagia and obesity in adult offspring, with the phenotype proposed to persist in later generations via differential DNA methylation of serotonin receptor signaling genes [10,12].

This paper is a hypothesis-driven narrative review. Relevant literature was identified through searches of PubMed and Google Scholar, with the final literature search conducted on 9 August 2026, using terms including prenatal nutrition, tryptophan, serotonin, hypothalamic development, epigenetic programming, Great Hunger, and obesity. No formal inclusion or exclusion criteria were applied. The Irish case is presented as a testable hypothesis integrating established biochemical mechanisms with historical and epidemiological evidence; it is not presented as a demonstration of a proven causal chain within the Irish population.

Therefore, direct mechanistic influences of dietary alterations during The Great Hunger likely led to a cascade that impacted obesity acutely and across immediate and possibly subsequent generations.

## 2. The Great Hunger: Characterization of Tryptophan Depletion

A complete understanding of the Great Hunger’s neurobiological outcome requires establishment of two facts: that the rural Irish population depended almost exclusively on the potato for dietary tryptophan and high-quality protein, and that this source was eliminated over multiple consecutive growing seasons. Both are well established in the historical and bioarcheological record.

The potato (*Solanum tuberosum*) provides an almost complete protein with a Digestible Indispensable Amino Acid Score (DIAAS) comparable to soy protein and substantially superior to the maize and wheat-based foods that replaced it during the starvation period [8]. At the consumption levels documented for the rural poor, the potato supplied adequate tryptophan to sustain serotonin synthesis and provided the pyridoxine needed as cofactor for tryptophan hydroxylase [13]. The foods substituted through relief provision, specifically Indian maize meal, were deficient in tryptophan relative to potato protein [14]. The Great Hunger therefore constituted a transition from a diet adequate in tryptophan to one that was chronically and severely deficient, sustained across seven growing seasons.

This depiction is supported directly by bioarcheological analysis. Beaumont and Montgomery [15] found nitrogen isotope signatures in bone collagen from skeletal remains from the Great Hunger period consistent with an abrupt and sustained withdrawal of a high-quality complete protein source. Geber et al. [14] detected corn residues in 93% of individuals from the Kilkenny Workhouse burial and potato residues in only 12%, confirming near-complete dietary substitution. Together, these data demonstrate the biological reality of the tryptophan specific nutritional collapse.

### 2.1. Tryptophan as the Rate-Limiting Substrate for Central Serotonin Synthesis

Serotonin (5-hydroxytryptamine, 5-HT) is synthesized from tryptophan in two steps. Tryptophan is first hydroxylated to 5-hydroxytryptophan (5-HTP) by tryptophan hydroxylase (TPH), the rate-limiting enzyme of the pathway, which requires tetrahydrobiopterin as cofactor and molecular oxygen. 5-HTP is then decarboxylated to 5-HT by aromatic L-amino acid decarboxylase (AADC), which requires pyridoxal phosphate (derived from pyridoxine, Vitamin B6) as cofactor [16]. Both TPH and AADC are expressed in central serotonergic raphe neurons and in enterochromaffin cells of the gastrointestinal mucosa, which produce approximately 95% of total body serotonin.

TPH activity is directly substrate limited under physiological conditions. TPH operates well below saturation at normal plasma tryptophan concentrations, and central 5-HT synthesis rate therefore increases and decreases proportionally with dietary tryptophan availability [17]. Experimental acute tryptophan depletion, achieved by administration of a tryptophan-free amino acid mixture that competitively reduces plasma tryptophan transport into the brain, produces measurable reductions in central serotonergic activity within hours, resulting in impairment of serotonin mediated appetite suppression, mood regulation, and sleep regulation [17]. Sustained dietary tryptophan deficiency across the period of fetal hypothalamic development would produce a chronic, severe reduction in fetal central serotonin synthesis at the developmental stage when serotonin is most critical for circuit formation, establishing it as the result of The Great Hunger.

The potato supply of pyridoxine (vitamin B6) is especially important from a neurobiological perspective. Pyridoxal phosphate is an obligate cofactor for AADC, and B6 deficiency specifically reduces the conversion of 5-HTP to 5-HT independently of tryptophan supply. The replacement of potatoes with B6-deficient maize meal led to a compounded disruption in serotonin synthesis, involving simultaneous depletion of both the substrate (tryptophan) and the cofactor (vitamin B6). Further, the potato contains both tryptophan and B6, making it uniquely suited as a serotonin precursor source, with its removal resulting in a compound biochemical deficit [8].

### 2.2. The Dual Role of Serotonin in Fetal Hypothalamic Development

In the adult brain, serotonin functions as a classical neurotransmitter, acting at postsynaptic receptors to modulate neuronal excitability and regulate appetite, mood, sleep, and cognition. During fetal development, however, 5-HT plays a broader role as a morphogen, regulating the development of brain circuitry that will then use monoaminergic, peptidergic, and other neurotransmitter systems in adulthood. This trophic serotonin function, referred to as the organizational role of 5-HT, runs through mechanisms independent of synaptic transmission and includes regulation of neuronal proliferation, migration, axonal branching, and synaptogenesis [9,18].

In the hypothalamus, 5-HT modulates the development of the arcuate nucleus (ARC) and the pathways connecting ARC neurons to downstream appetite regulating nuclei, including the paraventricular nucleus (PVN), dorsomedial hypothalamus (DMH), and lateral hypothalamic area (LHA). These projections are collectively forming the central melanocortin system and are the primary long-term regulators of energy homeostasis. Their structural organization is established during the second and third trimesters in humans and is highly dependent on the nutritional and hormonal environment present during this critical period [9,11]. Disruption of serotonin availability during this period therefore does not simply impair acute neurotransmitter function, but permanently alters the structure of the circuit that manages energy balance throughout adult life.

### 2.3. Arcuate Nucleus Structure: POMC, NPY/AgRP, and Serotonergic Control

The ARC contains two functionally antagonistic neuronal populations that serve as the primary afferent sensors of peripheral satiety and hunger signals. POMC neurons project to downstream nuclei and release alpha-melanocyte-stimulating hormone (alpha-MSH), a cleavage product of the POMC precursor peptide that acts at melanocortin-3 and melanocortin-4 receptors (MC3R, MC4R) to suppress food intake and increase energy expenditure. Agouti-related peptide and neuropeptide Y (AgRP/NPY) neurons act synergistically to promote feeding, releasing AgRP, a competitive antagonist at MC3R and MC4R, and NPY, which independently stimulates appetite, together generating a strong orexigenic signal [9,11].

Serotonin modulates this circuit primarily through 5-HT2CR, which is expressed at high density on ARC POMC neurons. 5-HT2CR is a Gq-coupled GPCR (G-protein coupled receptor) whose activation depolarizes POMC neurons, increasing alpha-MSH release and downstream MC4R-mediated satiety signaling. It also suppresses activity in AgRP/NPY neurons through POMC-mediated inhibitory interneuron pathways, amplifying the net anorexigenic signal. The role of 5-HT2C receptors in appetite regulation is supported by the finding that pharmacological 5-HT2C receptor agonists (e.g., lorcaserin, which was withdrawn from the US market in 2020 following a post-market cancer risk signal [19]; selective 5-HT2C agonists remain under active investigation) represent a mechanistically validated anti-obesity drug class, and that 5-HT2C receptor knockout mice exhibit hyperphagia and late onset obesity even when maintained on a normal chow diet [10].

The 5-HT2A receptor (5-HT2AR) plays a secondary and complementary role within the ARC. 5-HT2AR is expressed on a specific ARC neuronal population and also produces anorexigenic signals when activated, but through partially different downstream pathways than 5-HT2CR. The two receptors can compensate for each other under some conditions, but the compensation is incomplete. Mice with isolated 5-HT2CR deletion develop obesity despite intact 5-HT2AR signaling, proving the primacy of 5-HT2CR in serotonin mediated appetite suppression [10].

### 2.4. Serotonergic and Kynurenine Pathways

Most dietary tryptophan does not become serotonin. Approximately 90–95% is metabolized through the kynurenine pathway, leaving only a small fraction available for conversion to the neurotransmitter responsible for satiety regulation [20]. Understanding this competition matters for the obesity argument because the proportion routed toward serotonin is not stable. It responds to inflammatory signals, nutritional depletion, and metabolic stress in ways that directly affect hypothalamic appetite circuits.

The kynurenine pathway generates metabolites with opposing effects on neural function. Kynurenic acid acts as an NMDA receptor antagonist and carries neuroprotective properties, while quinolinic acid is an excitotoxic NMDA receptor agonist capable of inducing neuronal damage when present in excess [20,21]. Under inflammatory conditions, pro-inflammatory cytokines including interferon-gamma, interleukin-6, and tumor necrosis factor-alpha upregulate indoleamine 2,3-dioxygenase, the enzyme that initiates the kynurenine pathway. This effectively redirects tryptophan away from serotonin synthesis and toward kynurenine metabolite production [20,21,22]. Conditions of severe food insecurity, infection, and physiological stress are sufficient to trigger this shift, all of which were characteristic of the population that lived through The Great Hunger.

The metabolic consequences of this diversion are relevant to the obesity phenotype proposed here. Reduced serotonin availability directly weakens 5-HT2CR-mediated activation of arcuate nucleus POMC neurons, impairing the satiety signal that normally suppresses food intake and counters AgRP/NPY driven hunger. At the same time, increased kynurenine pathway output alters glutamatergic tone in hypothalamic circuits that regulate energy balance [20,22]. The result is a coordinated weakening of appetite suppression from two directions at once, with both effects operating on the same developmental circuitry during the prenatal window most sensitive to serotonin availability.

For starvation era fetuses, this dynamic was compounded by the direct substrate shortage caused by loss of the potato as a tryptophan source. The already depleted tryptophan pool would have faced additional pressure from IDO mediated catabolism driven by the infectious disease burden and physiological stress that characterized The Great Hunger. Both mechanisms result in the same outcome, less tryptophan reaching central serotonin synthesis at the period of hypothalamic circuit formation, and a greater proportion of what remained being converted into compounds that disrupt rather than support normal neural development. This pathway provides a second, mechanistically distinct route through which the nutritional deprivation of The Great Hunger could have produced the persistent obesity associated phenotype documented in subsequent generations [20,21,22].

### 2.5. Maternal Tryptophan Restriction, Receptor Dysregulation, and Offspring Obesity

The most direct experimental evidence linking maternal dietary tryptophan restriction to offspring hypothalamic serotonin receptor dysregulation and obesity was provided by Martin-Gronert et al. [10] in a study providing mechanistic relevance to the paper’s hypothesis. Pregnant rats were kept on a low-protein diet (8% versus 20% casein in controls) that simultaneously reduced tryptophan availability and overall protein quality, designed to model conditions in which the primary protein source had been removed and replaced with nutritionally inferior alternatives. This design shares structural similarities with the Great Hunger transition from potato to Indian maize meal, though the model used a low-protein diet rather than tryptophan-specific restriction.

Three significant neurobiological findings were reported. First, maternal low-protein diet elevated serum and placental 5-HT concentrations, with elevated 5-HT detected in fetal brain tissue at embryonic day 16.5. The authors proposed that sustained elevation of 5-HT during this developmental window drove receptor downregulation: chronic exposure to elevated serotonin caused compensatory reduction in hypothalamic 5-HT2CR expression, consistent with receptor desensitization. This mechanism was not directly demonstrated in the study. Importantly, this pathway differs from the one proposed for the Great Hunger, in which reduced tryptophan availability would limit fetal serotonin synthesis. In the Martin-Gronert model, fetal 5-HT is elevated rather than reduced, and the downstream receptor downregulation is the shared feature linking both scenarios to the same obesity phenotype. Second, hypothalamic 5-HT2CR expression was significantly reduced in the fetal, neonatal, and adult hypothalamus of offspring from low-protein dams, compared to controls matched for gestational age and postnatal nutrition. Third, hypothalamic 5-HT2AR mRNA was upregulated in offspring who had undergone rapid postnatal catch-up growth following intrauterine growth restriction, potentially as a compensatory mechanism, but this upregulation was insufficient to restore normal serotonin mediated appetite suppression.

The phenotypic consequence of 5-HT2C receptor down regulation in these animals was reduced sensitivity to serotonergic anorectic stimulation, as shown by a diminished suppression of feeding in response to a 5-HT2C receptor agonist, confirming the functional significance of the receptor-level change. Furthermore, offspring were predisposed to hyperphagia and diet-induced obesity in adulthood, even when maintained on a standard diet post-weaning, indicating that developmental receptor programming produced a lasting shift in the energy balance set point rather than a transient nutritional effect. Several limitations apply to this model: the experiment used rats not humans; a general low-protein diet was used rather than tryptophan-specific or B6-specific restriction; sex-specific effects were not fully characterized; and postnatal catch-up growth may have confounded the programming interpretation. Human confirmation of tryptophan-restricted hypothalamic 5-HT2C receptor changes during a comparable prenatal window is outstanding.

### 2.6. The Peripheral Gut–Brain Serotonin Axis and Its Nutritional Vulnerability

The functional significance of gut derived serotonin in appetite regulation is increasingly recognized as a contributor to famine-induced metabolic dysregulation [16]. Enterochromaffin cells lining the intestinal mucosa represent the largest serotonergic cell population in the body and release 5-HT in response to nutrient sensing, mechanical stimulation, and luminal chemical signals. It is important to note that peripheral serotonin does not freely cross the blood–brain barrier; maternal, placental, fetal intestinal, and central serotonergic compartments represent functionally distinct pools. Where this review refers to serotonin availability in fetal brain development, it refers specifically to de novo central synthesis from transplacentally delivered tryptophan, not to circulating maternal or gut-derived 5-HT. Intestinal 5-HT activates vagal afferent fibers that project to the nucleus tractus solitarius (NTS) and, via the dorsal vagal complex to hypothalamic appetite nuclei, forming a peripheral satiety signal that is integrated with the central melanocortin system [16]. Chronic dietary tryptophan deficiency reduces enterochromaffin serotonin synthesis and release, thereby impairing this peripheral satiety pathway. In the developing fetus, intestinal serotonin also influences gastrointestinal morphogenesis and the maturation of the enteric nervous system, and perinatal serotonin deficiency has been associated with persistent alterations in gut motility and nutrient absorption that may contribute to the metabolic phenotype observed in famine-exposed offspring [16].

## 3. Quantitative Reconstruction of Tryptophan Depletion During the Great Hunger

The preceding sections establish the biological mechanism through which maternal tryptophan restriction during the Great Hunger could have programmed offspring hypothalamic circuits toward obesity. What has not been established is the actual magnitude of that restriction. Prior accounts describe the potato as a tryptophan-containing food and note that its loss coincided with increased metabolic and psychiatric morbidity, but without quantifying how much tryptophan was available before the crisis, how much remained during it, or how long the deprivation continued after the acute years had passed. This section addresses that gap directly. Using primary dietary survey data from Clarkson and Crawford [23], crop acreage records from Ó Gráda [24], and nutritional composition values from the United States Department of Agriculture [25], a reconstruction of tryptophan availability is presented across four periods: the pre-1845 baseline, the crisis years of 1845 to 1852, the workhouse diet during 1848 to 1849, and the post-crisis recovery as documented in 1859. The reconstruction indicates that estimated tryptophan depletion was severe rather than moderate, that the mechanism responsible was not simple caloric restriction alone but also a specific biochemical redirection of available tryptophan away from serotonin synthesis, and that dietary recovery through the 1850s remained incomplete in a way that extended the window of suppressed maternal tryptophan availability well beyond the acute years of the crisis. These calculations are a theoretical reconstruction built from historical dietary surveys, crop acreage records, and modern food composition values. They estimate nutrient availability at the population level under stated assumptions, and they are not a direct measurement of maternal, fetal, or central serotonin status, none of which can be recovered from the historical record.

### 3.1. The Pre-1845 Nutritional Baseline

Clarkson and Crawford [23], drawing on Hawley’s 1839 field survey across 13 Poor Law unions in Munster, record average daily potato consumption by adult male laborers at 184 ounces per day, equivalent to 5216 g. At 27 mg of tryptophan per 100 g of boiled potato [25], potato alone supplied 1408 mg of tryptophan per day. Buttermilk, the standard daily supplement to the laboring diet, was recorded at 58 ounces per day in the same table, contributing a further 592 mg at 36 mg of tryptophan per 100 milliliters [25]. Total daily tryptophan intake from these two foods was 2000 mg per day.

The World Health Organization sets the adult minimum tryptophan requirement at 4 mg per kilogram of body weight per day, giving 280 mg per day for a 70 kg adult [26]. The pre-1845 laboring diet was supplying tryptophan at 714% of this minimum. The same diet provided 14.1 mg of vitamin B6 per day from potato alone at 0.27 mg per 100 g [25], representing 830% of the 1.7 mg per day recommended intake [27], and 25.6 mg of iron per day from the full diet [23], representing 320% of the 8 mg per day recommended daily intake [27]. Clarkson and Crawford [23] confirm that total dietary energy was 4868 kilocalories per day and protein 153.3 g per day. The pre-1845 diet was deficient only in vitamins A and D [23]. Every nutrient relevant to tryptophan hydroxylase function, the substrate (tryptophan), the B6 cofactor, and the iron cofactor, was present in large surplus. This baseline matters because it reveals that the fall triggered by the crisis was not from marginal adequacy to mild deficiency, but from a position of very large surplus to complete depletion.

### 3.2. The Collapse of Potato Supply and the Introduction of Indian Corn (1845–1852)

Ó Gráda [24] provides potato acreage data for each of the 32 Irish counties across the years of the crisis. Total acreage fell from 2,186,798 acres in 1845 to 274,134 acres in 1847, a loss of 87.5% across all counties. Applying this proportional reduction to the documented pre-1845 consumption of 5216 g per day reduces available potato to approximately 652 g per day, assuming even distribution, which historical records indicate did not occur. The western counties where laboring dependence on the potato was greatest experienced the steepest declines. Tryptophan from the reduced potato supply fell to 176 mg per day.

Indian corn was imported as the primary replacement food. Clarkson and Crawford [23], using Crawford’s analysis of Irish Customs records, document Indian corn and meal imports at an annual average of 5,833,014 hundredweight across 1845 to 1849. Distributed across an estimated population of 7.5 million, this amounts to approximately 108 g of dry cornmeal per person per day, a figure derived directly from import records rather than from an estimate. At 70 mg of tryptophan per 100 g of dry cornmeal [25], the corn ration contributed 76 mg of tryptophan per day. Combined with the 176 mg from reduced potato, gross tryptophan from the relief diet was 252 mg per day, already below the WHO minimum of 280 mg before any additional depletion mechanism applied.

### 3.3. Niacin Diversion: Why the Substitute Food Reduced Tryptophan Further

The substitution of Indian corn for potato did not simply reduce tryptophan through caloric replacement. It introduced a competing biochemical demand that consumed much of the tryptophan still available. Tryptophan serves two metabolic functions in the body. It is the only dietary precursor for serotonin, and it is also the body’s reserve source of niacin (vitamin B3) when dietary niacin is insufficient. The kynurenine pathway converts tryptophan to niacin at a fixed ratio of 60 mg of tryptophan per 1 mg of niacin produced [27]. When dietary niacin is adequate, this conversion does not occur at scale and tryptophan is available for serotonin synthesis. When dietary niacin is deficient, the conversion becomes a metabolic priority and serotonin synthesis loses its substrate. This process is referred to here as niacin diversion.

Corn contains niacin, but in a chemically bound form called niacytin that the human gut cannot absorb. The release of bound niacin from corn requires alkaline processing, known as nixtamalization, a technique developed in Mesoamerica that underlies traditional tortilla and hominy preparation. The Irish population had no knowledge of this process, and the corn was distributed as raw ground meal. As a result, bioavailable niacin from 108 g of unprocessed Indian corn was only 1.84 mg per day, at 1.7 mg per 100 g [25], against an adult daily requirement of 14 mg [27]. The niacin deficit was 12.16 mg per day. At the 60:1 conversion ratio, covering that deficit required diverting 730 mg of tryptophan from serotonin synthesis toward niacin production. The gross tryptophan supply from the relief diet was 252 mg. The niacin demand consumed 730 mg. Net tryptophan available for serotonin synthesis after niacin diversion was negative 478 mg. A negative value of this kind is an accounting outcome rather than a physiological state, and it indicates that the estimated niacin demand exceeded the entire estimated tryptophan supply, so that on these assumptions little would have remained for serotonin synthesis. In practice such a shortfall would have been met partly through tissue protein catabolism and partly through unmet niacin requirement, the latter expressed clinically as pellagra. This is the same mechanism that drove epidemic pellagra across the American South and parts of southern Europe wherever populations subsisted on unprocessed corn. Crawford [23] noted pellagra-like presentations in Ireland during and after the years of the Great Hunger, likely masked by the concurrent prevalence of fever and dysentery.

The pre-1845 diet did not create this problem because the potato provides bioavailable niacin freely, without any tryptophan cost. The switch from potato to unprocessed Indian corn was therefore a switch from a food that protected tryptophan for serotonin synthesis to one that actively redirected available tryptophan toward niacin compensation. The result was not simply less tryptophan but a biochemical mechanism that converted the substitute food’s very presence into a driver of further serotonin precursor depletion.

### 3.4. IDO Activation from Epidemic Disease: A Second Independent Depletion Mechanism

Epidemic disease during the Great Hunger years added a second route of tryptophan depletion that operated independently of dietary intake. Clarkson and Crawford [23] document mortality from infectious diseases across Ireland from 1844 to 1851. In 1847, typhus and relapsing fever killed 57,095 people, accounting for 46.4% of all recorded disease deaths. Dysentery and diarrhea accounted for 36,464 deaths, representing 29.6% of disease mortality. Together these two categories, both highly inflammatory, accounted for 76.0% of all 1847 disease deaths, from a total of 123,172 disease deaths in that year.

Typhus and dysentery both strongly upregulate indoleamine 2,3-dioxygenase (IDO), the enzyme that initiates the kynurenine pathway by converting tryptophan to kynurenine in response to immune activation. As described above, IDO is activated by pro-inflammatory cytokines including interferon-gamma, interleukin-6, and tumor necrosis factor-alpha [22]. Dantzer and colleagues [28] documented that IDO activation during acute inflammatory states reduces circulating tryptophan by 30 to 50%, independently of dietary intake. Applying the lower bound of 30% to the 252 mg gross tryptophan supply removes an additional 76 mg before niacin diversion is applied, resulting in an estimated net tryptophan availability under compound conditions of negative 553 mg on the same accounting basis. For pregnant women who were simultaneously undernourished, consuming unnixtamalized corn, and surviving epidemic fever or dysentery, all three mechanisms operated in parallel. Dietary depletion, niacin diversion, and IDO activation each reduced tryptophan through a different pathway, and each was independently severe enough to impair fetal serotonin synthesis on its own.

### 3.5. Dietary Recovery After the Crisis and the Extended Exposure Window (1859)

The nutritional suppression of tryptophan availability did not end when the acute crisis did. Clarkson and Crawford [23] provide dietary data for 1859, a decade after the worst years of the Great Hunger. Potato consumption had partially recovered to 61 ounces per day, equivalent to 1729 g, contributing 467 mg of tryptophan. Buttermilk had recovered to 38 ounces per day, contributing 388 mg. Indian corn at 18 ounces per day, equivalent to 510 g, had become a permanent fixture in the post-crisis Irish diet, contributing 357 mg of tryptophan. Gross tryptophan from the 1859 diet was 1212 mg, substantially higher than the crisis-period figure.

Indian corn in 1859 was still not being nixtamalized. At 510 g per day, available niacin was only 8.67 mg against the 14 mg requirement, leaving a deficit of 5.33 mg and diverting 320 mg of tryptophan toward niacin synthesis. Net tryptophan available for serotonin synthesis in 1859 was 892 mg, representing 319% of the WHO minimum. This appears to represent recovery, but against the pre-1845 baseline of 2000 mg, the 1859 diet was providing only 45% of pre-crisis serotonin precursor availability. The permanent incorporation of Indian corn into the Irish diet after the Great Hunger kept a niacin diversion mechanism running throughout the 1850s, suppressing net tryptophan below half the pre-1845 level for the entire decade following the acute crisis.

This finding has direct implications for the scope of the epigenetic effects this paper proposes. If the relevant exposure were limited to the acute crisis years of 1845 to 1852, the affected birth cohorts would be confined to that window. The 1859 data indicate that the nutritional conditions depressing maternal tryptophan availability and, by extension, fetal serotonin synthesis persisted through at least 1859. Mothers giving birth throughout the 1850s were operating on diets in which net tryptophan remained at less than half the pre-1845 baseline because of ongoing niacin diversion from corn. Children born across this entire post-crisis decade were developing their hypothalamic serotonin receptor systems under conditions of suppressed maternal tryptophan, not only those born at the crisis peak. This extended exposure window is consistent with the persistence of elevated metabolic risk across multiple Irish birth cohorts and with the period of elevated psychiatric admissions documented by Walsh [29] between 1860 and 1875.

### 3.6. Mechanistic Implications of the Quantitative Reconstruction

The quantitative findings reported above speak directly to the question of whether the Great Hunger produced nutritional conditions sufficient to disrupt fetal hypothalamic serotonin receptor development at the scale that the present paper’s mechanism requires. They do so in three respects. First, the pre-1845 Irish laboring diet provided tryptophan, vitamin B6, and iron, the three inputs required for tryptophan hydroxylase activity and serotonin synthesis, at levels far above any plausible threshold of adequacy. There was no pre-existing marginal deficiency to contend with. Second, the transition to a relief diet based on Indian corn did not simply reduce tryptophan intake. It introduced niacin diversion, a biochemical mechanism by which the substitute food itself competed for the tryptophan that remained, redirecting the substrate that would otherwise have supported serotonin synthesis toward niacin compensation. This is a qualitatively different insult from caloric restriction alone. Third, the incomplete dietary recovery documented in 1859, with net tryptophan at 45% of the pre-1845 baseline due to the permanent incorporation of Indian corn, extended the window of suppressed maternal tryptophan availability across an entire decade of birth cohorts beyond the crisis years.

The fetal hypothalamic window during which 5-HT2CR expression is established and serotonergic appetite circuits are organized corresponds to the second and third trimesters of human gestation [9,11]. For fetuses exposed to these conditions during that window, the developmental receptor programming described by Martin-Gronert and colleagues [10] would have operated under severe substrate limitation. The combination of negligible estimated net tryptophan, impaired B6 cofactor status, and IDO-mediated redirection of residual tryptophan toward the kynurenine pathway rather than serotonin synthesis describes a scenario in which the principal routes to adequate fetal serotonin would have been compromised simultaneously. This remains an inference from reconstructed population-level intake rather than a measured fetal exposure. This reconstruction provides the quantitative grounding that the epidemiological and epigenetic evidence presented in the sections that follow requires.

## 4. Epigenetic Mechanisms of Intergenerational Transmission

### 4.1. DNA Methylation as the Molecular Substrate of Developmental Programming

The persistence of famine associated metabolic risk across multiple generations implies a mechanism capable of transmitting environmentally induced phenotypic changes within a timescale too short to be explained by genetic mutation. DNA methylation of CpG dinucleotides in gene regulatory regions represents the most extensively documented mechanism in prenatal nutritional programming. Methylation of promoter-proximal CpG sites can inhibit transcription factor binding and recruit methyl-CpG binding proteins, promoting condensed chromatin states associated with reduced gene expression. These epigenetic marks are established during the process of de novo methylation that follows embryonic implantation, depend on the availability of methyl group donors such as S-adenosylmethionine (SAM), and are influenced by dietary methionine, folate, and B vitamins. As a result, they are responsive to the broader nutritional environment in ways that can alter the transcriptional output of metabolic regulatory genes [30,31].

Relevant to the serotonin hypothesis, the promoter regions of 5-HT receptor genes contain CpG islands whose methylation status influences receptor expression levels. Differential methylation of these regions, established during the prenatal programming window, provides a plausible molecular mechanism by which maternal tryptophan deficiency induced alterations in fetal serotonergic signaling may be encoded as stable changes in receptor gene expression, rather than fully normalizing after postnatal nutritional recovery.

Some methylation patterns established during embryonic development may persist due to incomplete erasure during epigenetic reprogramming of primordial germ cells (PGCs), allowing for potential transmission across generations. However, the extent of transgenerational epigenetic inheritance in humans remains uncertain. The degree to which famine-associated methylation changes are transmitted through the germline, rather than arising from repeated in utero exposures across generations, is still not clear. Regardless, reports of multigenerational effects on obesity and psychiatric outcomes following famine exposure are consistent with at least a partial contribution of germline transmission [32].

### 4.2. IGF2 DMR Methylation and the Dutch Hunger Winter Cohort

Foundational human evidence that prenatal famine produces persistent, measurable changes in DNA methylation was provided by Heijmans et al. [30], who compared methylation at the IGF2 differentially methylated region (DMR) in 60 individuals exposed to the Dutch Hunger Winter during the periconceptional period and their unexposed same-sex siblings. The IGF2 DMR is an imprinted regulatory region in which methylation governs monoallelic expression of insulin-like growth factor 2, a key regulator of fetal growth and postnatal metabolic programming. Individuals exposed to famine early in development exhibited 5.2% lower methylation at this region (0.488 versus 0.515; *p* = 0.000059), corresponding to a 0.48 standard deviation difference relative to controls, with this effect persisting for more than six decades. This difference was specific to periconceptional exposure and showed no association with sex or current metabolic status, supporting an origin in the early embryonic wave of de novo methylation.

Tobi et al. [31] extended these findings using a genome-wide approach and identified differential methylation across 181 CpG sites in the Dutch Hunger Winter cohort. These sites were enriched in genes involved in lipid metabolism, insulin signaling, growth, and development. The span of this methylation pattern suggests a coordinated response to early nutritional restriction that affects multiple gene networks rather than a single locus. Van Dijk et al. [33] further supported this pattern in a systematic review, concluding that DNA methylation at energy balance regulatory loci provides a consistent molecular link between the prenatal nutritional environment and adult metabolic phenotype across independent human cohorts.

### 4.3. Serotonin Receptor Gene Methylation Following Prenatal Famine: Direct Evidence

The epigenetic evidence most directly relevant to the present hypothesis is provided by Zeng et al. [12], who conducted genome-wide DNA methylation profiling in adults prenatally exposed to the Great Chinese Famine of 1959 to 1961 and compared them with demographically matched unexposed controls. Their primary analysis identified 40 differentially methylated positions (DMPs) that met genome-wide significance thresholds. Subsequent pathway enrichment analysis of these DMPs, along with differentially methylated regions (DMRs), revealed significant enrichment in the serotonin receptor signaling pathway. This finding provides evidence, based on whole-blood DNA methylation, that prenatal nutritional deprivation is associated with differential methylation in the serotonin receptor signaling pathway, particularly in females. The study did not measure hypothalamic receptor expression, serotonergic signaling, or obesity outcomes directly, and the methylation was measured in blood rather than brain tissue. Reduced methylation at these loci should not be interpreted automatically as reduced receptor expression; its functional significance depends on genomic location and regulatory context.

Zeng et al. [12] propose that prenatal nutritional restriction reduces tryptophan availability and, in turn, serotonin receptor stimulation during a critical developmental window when receptor gene methylation patterns are established. This process is thought to shift CpG methylation at serotonin receptor promoter regions, leading to reduced receptor expression in the adult brain. This interpretation aligns with findings from the experimental model of Martin-Gronert et al. [10], offering a mechanistic explanation for the observed downregulation of 5-HT2C receptors in offspring of tryptophan-restricted dams. The replication of this pattern across distinct famine populations, including a Chinese cohort reliant on rice-based diets and an Irish population dependent on potatoes, supports the idea of a conserved biological response to tryptophan deficiency rather than a population specific epigenetic effect.

### 4.4. Neuropsychiatric Co-Morbidity as Mechanistic Confirmation

If dysregulation of hypothalamic serotonin signaling underlies famine induced metabolic risk, famine exposed populations would also be expected to show elevated rates of serotonin related neuropsychiatric conditions independent of metabolic outcomes, given the shared involvement of serotonergic circuits in both appetite regulation and affective processing. Eichenauer and Ehlert [32] conducted a systematic review examining associations among prenatal famine exposure, DNA methylation, and mental health outcomes across multiple cohorts. Their analysis identified increased rates of schizophrenia spectrum disorders and elevated depressive symptoms in individuals exposed to famine prenatally, with DNA methylation proposed as a mediating mechanism. The occurrence of both metabolic syndrome and affective disorders in these populations is consistent with a shared disruption in serotonergic circuitry that may affect both hypothalamic appetite regulation and limbic and prefrontal systems involved in emotional processing, instead of independent pathological effects of famine exposure.

### 4.5. Autophagy-Induced Chromatin Remodeling as a Second Epigenetic Channel

The DNA methylation mechanisms described above operate within a broader cellular response to amino acid starvation that has been characterized as a source of heritable epigenetic change independent of the developmental de novo methylation window. González-Rodríguez et al. [34] review evidence that starvation induced autophagy, the cell’s conserved catabolic response to nutrient shortage, activated upon mTORC1 inactivation when amino acids are limiting, is directly associated with heritable chromatin modifications. When amino acids are depleted, mTORC1 is inactivated and autophagy is induced. Within the nucleus, the autophagy induction cascade upregulates expression of DNMT3A (DNA methyltransferase 3A) via a ULK3-GLI1 signaling axis, leading to methylation of autophagy related gene promoters including MAP1LC3 (Figure 1). Importantly, brief autophagic stimuli including amino acid starvation are enough to produce persistent DNMT3A mediated methylation changes that outlast the starvation event itself. In zebrafish, transient autophagy induction produced persistent downregulation of autophagy related gene expression into adulthood, and in murine lung tissue, the neonatal starvation period produced sustained changes in Map1lc3b expression that persisted across longitudinal observation. Together, these findings represent what González-Rodríguez et al. [34] termed an “epigenetic memory of autophagy”.

The human evidence for this mechanism in the context of famine is provided by the Chinese Suihua cohort analyzed by Jiang et al. [35] and reviewed by González-Rodríguez et al. [34]. Blood DNA methylome analysis of 138 subjects across two generations identified 961 and 503 differentially methylated sites in the F1 and F2 generations respectively between famine exposed and non-exposed groups (Figure 1). Of these, 19 differentially methylated sites were conserved across both generations, including loci in CUX1, PPARGC1A, ELMO1, and AGTR1, all of which regulate autophagy. The conservation of autophagy-regulatory gene methylation across two generations in a famine cohort is consistent with starvation-induced autophagy producing potentially heritable epigenetic marks through the DNMT3A pathway, though shared environment and parental physiology remain alternative explanations, operating in parallel to the serotonin receptor gene methylation documented by Zeng et al. [12]. The PPARGC1A locus encodes PGC-1α, a regulator of mitochondrial biogenesis and energy metabolism. Its differential methylation in both F1 and F2 famine-exposed individuals is consistent with possible heritable reprogramming of metabolic regulation, though F2 observations alone do not establish germline-mediated transgenerational transmission, as direct gestational exposure of fetal germ cells may account for F2 effects.

González-Rodríguez et al. [34] also review histone modification evidence. Autophagy induction is associated with changes in H3K4me3, H3K9me3, H3K27me3, H4K16ac, and H4K20me3, several of which are established transgenerational inheritance marks in invertebrates and are being documented in vertebrate germ cells. H3K9me3 and H3K27me3 are repressive marks that are altered during starvation induced autophagy, and may propagate through the germline in some model organisms, which has been proposed as a histone based epigenetic inheritance channel alongside DNA methylation. The mechanism is not tryptophan specific and is triggered by amino acid starvation generally. The Great Hunger’s seven-year duration and the specific removal of both the substrate and cofactor for serotonin synthesis would have made the autophagic response in fetal cells during the Great Hunger both prolonged and biochemically specific, driving DNMT3A-mediated methylation at energy metabolism and serotonin receptor gene loci together. The proposed result in offspring would be a genome epigenetically altered for chronic nutrient scarcity, with elevated appetite threshold, altered energy metabolism, and reduced serotonergic satiety signaling, developing an elevated risk for obesity that is consistent with Ireland’s current position at the upper end of European obesity statistics.

## 5. Cohort Epidemiology: Dutch Hunger Winter and Great Chinese Famine

### 5.1. Dutch Hunger Winter: Trimester-Specific Developmental Programming of Obesity

The cohorts reviewed below involve different nutritional exposures, durations, genetic backgrounds, and study designs. Their convergence supports the broader developmental-origins hypothesis but does not specifically validate a tryptophan-serotonin mechanism in each population. Evidence from these cohorts should be understood at five levels: (a) association between prenatal exposure and later metabolic outcomes; (b) persistent epigenetic differences following prenatal deprivation; (c) altered serotonin-related methylation in peripheral blood; (d) changes in hypothalamic receptor expression in animal models; and (e) evidence of germline transmission. The Irish case currently has evidence at levels (a) and (d), whereas levels (b), (c), and (e) remain untested in an Irish population.

The Dutch Hunger Winter cohort, which resulted from the Nazi blockade of the western Netherlands between November 1944 and May 1945, provides one of the most well documented human datasets for studying the developmental programming of adult obesity. Because the famine had a clearly defined beginning and end, and because detailed conscription and medical records are available, researchers can determine the timing of exposure during gestation with a high level of precision. This makes it possible to examine trimester specific effects in a way that is not possible in less clearly defined famine events.

Ravelli et al. [36] reported that men who were exposed to famine during the first half of pregnancy had significantly higher rates of obesity at military induction at age 18–20. In contrast, those exposed during the third trimester showed a slight but non-significant reduction in obesity rates. This pattern supports the developmental programming model, since hypothalamic appetite circuits are formed during early to mid-gestation. Nutritional restriction during this period likely disrupts the development of serotonergic systems involved in long-term appetite regulation. Ravelli et al. [37] later showed that this increased risk of obesity persisted into adulthood, remaining present at age 50 despite decades of normal nutrition and differences in adult lifestyle.

Roseboom et al. [38] further expanded on these findings by showing that prenatal famine exposure was associated not only with increased adiposity, but also with a broader metabolic phenotype, including central fat accumulation, insulin resistance, dyslipidemia, and increased cardiovascular risk. These results suggest that famine exposure affects the regulation of energy balance at a systems level, rather than just increasing fat accumulation.

### 5.2. Great Chinese Famine: Dose Response Evidence and Epigenetic Replication

The Great Chinese Famine of 1959 to 1961, which affected an estimated 15 to 30 million people, provides large scale epidemiological evidence that extends findings from the Dutch Hunger Winter to a genetically and culturally distinct population. An important strength of this cohort is the variation in famine severity across regions, which allows for dose response analyses. Li et al. [39] reported significantly higher rates of metabolic syndrome in adults who were exposed to famine prenatally in a cohort of more than 7000 participants. Similarly, Liu et al. [40] found a dose-dependent relationship between early life famine exposure and abdominal obesity in a nationally representative sample of 18,984 adults, with fetal exposure associated with increased obesity risk. This pattern is consistent with a developmental window during which hypothalamic systems are particularly vulnerable. Huang et al. [41] provided additional support for these findings in a separate large Chinese sample. Additionally, Lumey et al. [42] combined evidence across multiple international famine cohorts and showed that the link between prenatal famine exposure and adult obesity is reproducible across populations with different diets, genetic backgrounds, and socioeconomic conditions.

This cohort is especially relevant to the present review because the epigenetic analysis by Zeng et al. [12], which identified altered methylation in serotonin receptor signaling pathways, was conducted in this population. The occurrence of increased metabolic risk and serotonin related epigenetic changes within the same cohort provides one of the strongest human examples of the proposed mechanism; prenatal nutritional stress leading to altered serotonin signaling resulting in long-term metabolic outcomes.

### 5.3. Holodomor (Ukraine, 1932–1933): A Third Independent Cohort

The Holodomor (1932–1933), a Soviet engineered famine that resulted in the deaths of approximately four million Ukrainians, offers a third independent cohort in which prenatal exposure has been linked to adult metabolic disease on a population scale. Lumey et al. [43] analyzed data from the Ukrainian national diabetes register between 2000 and 2008, which included 128,225 cases of type 2 diabetes, alongside birth records for over 10 million individuals born in Soviet Ukraine between 1930 and 1939. Individuals born between January and June 1934, who were conceived during the peak of the famine in early 1933, showed a more than two-fold increase in type 2 diabetes risk compared to those born outside the famine period or in unaffected regions (OR 2.21; 95% CI 2.00 to 2.45). This effect followed a clear dose–response pattern, with higher risk seen in regions that experienced more severe famine, while no increase was seen in territories that were not exposed.

Li, Ó Gráda, and Lumey [44] combined findings from the Ukrainian, Dutch, and Chinese famine cohorts to estimate the population level burden of famine related type 2 diabetes. Their analysis suggested approximately 21,000 additional cases linked to the Holodomor, 400 cases linked to the Dutch Hunger Winter, and 0.9 million cases associated with the Chinese Famine. The consistency of these findings across three distinct populations supports the conclusion that prenatal nutritional restriction is a reproducible driver of adult metabolic disease. The Holodomor data are particularly compelling because the famine was time limited and varied geographically in severity, creating a natural experiment that helps reduce confounding factors related to genetics or long-term environmental conditions.

### 5.4. Great Finnish Famine: Reproductive Costs of Famine Exposure

The Great Finnish Famine of 1866–1868 killed approximately 110,000 people and provides evidence that famine exposure carries measurable physiological costs beyond metabolic programming. Women exposed to the famine while reproductively active showed a reduction in life expectancy of approximately 0.5 years per child, a cost not observed in women unexposed to the famine or exposed during development or post reproductive life [45,46]. Early life nutritional status in this population also directly shaped both mortality risk and reproductive success when individuals were later exposed to famine conditions, suggesting that the consequences of early deprivation extend beyond the immediate developmental window [47]. The Finnish data therefore add a further dimension to the relationship between famine and health, demonstrating that nutritional deprivation during reproduction carries costs for maternal longevity that are independent of offspring metabolic outcomes.

## 6. Palestine: Contemporary Food Insecurity as a Testable Context

The serotonergic developmental programming hypothesis generates a specific prediction: populations experiencing sustained multigenerational food insecurity should show elevated rates of overweight and obesity even in the absence of a single acute historical famine, if maternal tryptophan availability is chronically suppressed during critical gestational windows. Palestinian communities in Gaza and the West Bank document a consistent co-occurrence of household food insecurity and elevated rates of overweight and obesity across multiple demographic groups [48,49,50], a pattern consistent with this prediction. No longitudinal developmental programming data, methylation analyses, or receptor expression measurements are currently available in this population, and the present review does not treat these cross-sectional prevalence findings as epidemiological evidence for the proposed mechanism. They are noted here as identifying a contemporary setting in which the hypothesis could be directly tested through a prospective birth cohort study linking maternal nutritional status, fetal serotonin availability, epigenetic markers at serotonin receptor loci, and adult metabolic outcomes across generations.

## 7. Discussion

The evidence presented in this review is consistent with a proposed neurobiological explanation linking The Great Hunger to contemporary patterns of obesity. This model begins with the starvation due to destruction of the potato, which the present reconstruction indicates produced a sustained reduction in dietary tryptophan [13,14,15]. Because tryptophan is the primary precursor for serotonin, reduced availability limits serotonin synthesis [16]. During prenatal development, this reduction in serotonin may disrupt the normal maturation of hypothalamic appetite circuits, particularly the development of 5-HT2C receptor expression on pro-opiomelanocortin (POMC) neurons in the arcuate nucleus (ARC) [9,10].

Disruption at this stage has lasting consequences. Reduced 5-HT2C receptor expression is associated with increased food intake and obesity in adulthood, even under normal dietary conditions [10]. These changes may be stabilized through differential DNA methylation of genes involved in serotonin receptor signaling [12], allowing them to persist over time, and potentially across generations if epigenetic reprogramming during germ cell development is incomplete, though germline transmission has not been established for this pathway. In this way, a short-term nutritional disruption is converted into a lasting change in energy balance regulation.

At a population level, this mechanistic framework aligns with epidemiological evidence showing increased obesity risk following prenatal famine exposure across multiple independent cohorts [36,38,41] (Table 1). Although no single study demonstrates the entire pathway within the Irish population, the combination of mechanistic, molecular, and epidemiological findings across different contexts supports a biologically plausible explanation. The Great Hunger is particularly relevant within this framework because its nutritional profile was characterized by a pronounced reduction in tryptophan, making it consistent with a serotonergic mechanism of developmental programming.

This model also extends the Thrifty Phenotype Hypothesis proposed by Hales and Barker [51]. While that framework established that prenatal undernutrition can increase the risk of metabolic disease, it did not identify the specific biological pathway through which this programming occurs. The serotonergic model proposed here provides a potential mechanism, with reduced 5-HT2C receptor signaling in arcuate POMC neurons offering a likely basis for a long-term shift in energy balance.

Importantly, this model extends beyond metabolic outcomes. Famine exposed populations also show elevated rates of depression, anxiety, and schizophrenia spectrum disorders, patterns that are not accounted for by the original Thrifty Phenotype framework [32]. Because serotonergic systems are involved in both appetite regulation and affective processing, developmental disruption of these pathways would be expected to affect both areas. The occurrence of metabolic and neuropsychiatric conditions in famine exposed populations therefore supports the interpretation of a shared underlying mechanism, rather than separate consequences of nutritional deprivation.

The proposed serotonergic mechanism does not exclude alternative explanations for the metabolic and psychiatric outcomes observed following prenatal nutritional deprivation. Total energy and protein deficiency, independent of specific tryptophan depletion, can alter fetal growth trajectories and metabolic set points through glucocorticoid-mediated programming of the hypothalamic–pituitary–adrenal axis. Maternal psychological stress during the Great Hunger would have elevated cortisol independently of nutritional status. Epidemic infectious disease caused placental dysfunction and intrauterine growth restriction through mechanisms independent of tryptophan availability. Postnatal catch-up growth following intrauterine restriction is itself a documented driver of adult obesity and metabolic syndrome. Socioeconomic transmission of poverty, dietary habits, and stress across generations provides a non-epigenetic route through which consequences could persist. Population selection effects caused by famine mortality and mass emigration altered the genetic composition of the surviving population in ways that could have contributed to altered metabolic risk independently of developmental programming. The serotonergic hypothesis should be evaluated alongside these mechanisms rather than treated as a complete account.

It is also important to acknowledge that general energy deprivation, independent of tryptophan restriction, is itself a central feature of the Great Hunger and produces distinct epigenetic consequences. Famine is fundamentally characterized by profound caloric deficit, and this alone is sufficient to alter the fetal epigenome through pathways that do not require tryptophan as a mediator. Heijmans and colleagues [30] demonstrated that periconceptional exposure to the Dutch Hunger Winter produced 5.2% lower IGF2 DMR methylation in exposed individuals compared to their unexposed same-sex siblings, an effect that persisted for more than six decades and was specific to early gestational timing. Critically, this IGF2 hypomethylation reflects the consequences of general prenatal nutritional deprivation irrespective of tryptophan status, confirming that the serotonergic mechanism proposed in the present review represents one component of a broader epigenetic response to severe prenatal undernutrition rather than the only route through which the Great Hunger could have exerted lasting biological effects on subsequent generations.

The serotonergic model does not predict uniform psychiatric outcomes across all famine exposed populations. A qualitative study of adults born during the 1983 Ghana food crisis found that, alongside cognitive and socio-emotional vulnerabilities, famine exposure was associated with heightened prosocial traits including empathy, cooperation, and community solidarity [52]. These outcomes likely reflect both the nutritional profile of the Ghanaian crisis, which differed from the Great Hunger’s specific tryptophan depletion, and the role of cultural and kinship buffers in shaping downstream psychosocial development. The neuropsychiatric consequences of famine exposure are therefore shaped by its nutritional specificity, not simply its severity.

### Limitations

The primary limitation of this review is the duration between The Great Hunger and the emergence of molecular and epigenomic techniques. As a result, conclusions regarding the Irish case are necessarily inferential and based on converging evidence from other famine exposed populations. The finding by Zeng et al. [12] linking famine exposure to altered methylation in serotonin receptor pathways is currently based on a single cohort and would benefit from replication, particularly in the Dutch Hunger Winter population, where extensive epigenetic and metabolic data are already available. In addition, the experimental model by Martin-Gronert et al. [10] was conducted in rats and requires further research in human systems, including confirmation of the timing and extent of 5-HT2C receptor changes in the developing human hypothalamus.

Bioarcheological evidence supports the presence of protein deprivation during the Great Hunger but cannot directly quantify tryptophan availability. Furthermore, it is not currently possible to determine what proportion of modern Irish obesity prevalence can be attributed specifically to epigenetic inheritance from famine exposure, as this would require longitudinal data linking ancestry, methylation patterns, and metabolic outcomes. Contemporary analyses, such as Donovan and McNulty [2], emphasize that obesity in Ireland arises from a combination of dietary, environmental, socioeconomic, and genetic factors. The mechanism proposed here should therefore be understood as one contributing biological aspect within a broader framework instead of a replacement for established explanations. Further, fetal tryptophan availability should be examined in further detail. Maternal tryptophan restriction reduces 5-HT2CR expression as discussed [10,12]. However, work should be examined regarding fetal tryptophan availability [53].

## 8. Conclusions

The Great Hunger of 1845 to 1852 is reconstructed here as a population-scale, seven-year depletion of dietary tryptophan through the catastrophic failure of the potato monoculture, which would have produced prolonged substrate limitation of serotonin synthesis in a population of developing fetuses across multiple consecutive birth cohorts. The experimental evidence reviewed here indicates that, in a rat model, maternal dietary protein restriction is associated with persistent downregulation of hypothalamic 5-HT2C receptor expression on arcuate nucleus POMC neurons, impairing serotonin mediated satiety signaling and predisposing offspring to hyperphagia and diet induced obesity [10]. This receptor-level programming may be stabilized via differential DNA methylation of serotonin receptor signaling pathway genes, as suggested by findings in a human famine cohort reported by Zeng et al. [12]. The epidemiological consequences of prenatal famine induced metabolic programming are documented across the Dutch Hunger Winter and Chinese Famine cohorts, indicating a population level obesity risk elevation consistent across the lifespan [36,38,41]. Ireland’s position at the upper end of European obesity rankings is consistent with this hypothesis, though the contribution of the proposed mechanism relative to contemporary dietary, socioeconomic, and environmental factors has not been quantified, and the marked increase in Irish obesity during recent decades requires reconciliation with a supposedly long-standing inherited predisposition, and the specific tryptophan depleting nature of the Great Hunger makes the serotonergic programming pathway particularly relevant to the Irish case given the specific pre-1845 dependence on a single tryptophan source.

## Figures and Tables

**Figure 1:**
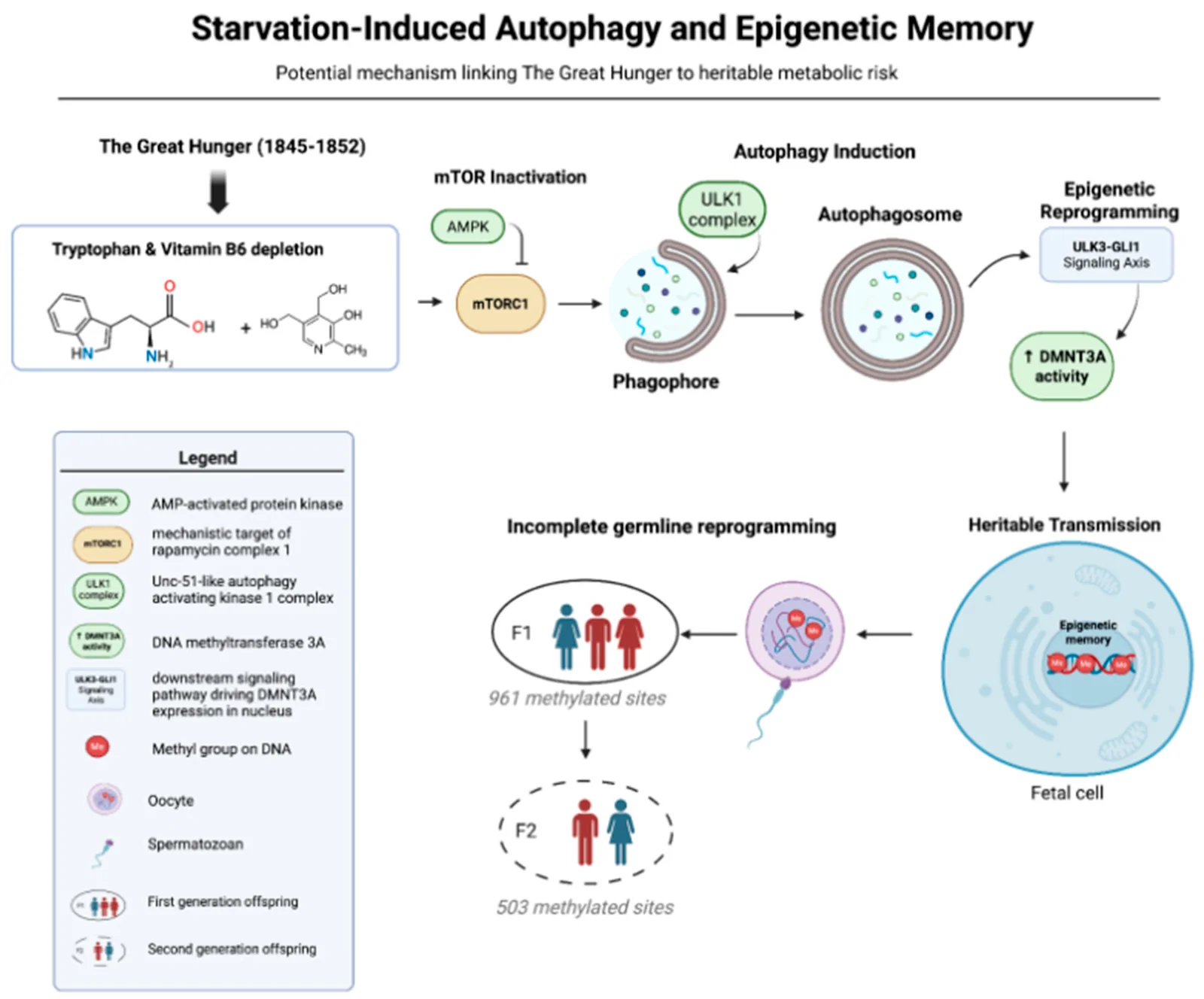
Starvation-induced autophagy and epigenetic memory in the context of the Great Hunger (1845–1852). Potential amino acid depletion following the collapse of the potato crop may. have inactivated mTORC1 and induced autophagy, potentially triggering DNMT3A upregulation via the ULK3-GLI1 signaling axis and methylation of gene promoters including MAP1LC3, PPARGC1A, and AGTR1. These modifications could possibly constitute an epigenetic memory of autophagy that outlasts the starvation event and may be transmitted across generations. Blood DNA methylome analysis of the Chinese Suihua famine cohort identified 961 differentially methylated sites in F1 offspring and 503 in F2 offspring, with 19 conserved across both generations [34,35]. Whether such marks are transmitted through the germline in humans has not been established, and the application of this pathway to the Great Hunger is hypothetical, since the experimental and cohort evidence shown is drawn from non-Irish models and populations. For further details on the proposed mechanisms, see [31,34].

**Table 1:** Evidence supporting prenatal nutritional disruption in metabolic programming via serotonergic pathways. Evidence is organized into five sections of increasing inferential specificity: famine metabolic outcome associations; persistent epigenetic differences; serotonin related methylation in peripheral blood; hypothalamic receptor expression in animal models; and germline transmission. The Irish case has evidence only in the first and fourth sections. Rows represent individual cohorts or experimental models. Major Limitation identifies the most consequential constraint on each claim.

Summary of Evidence Across Five Levels Supporting a Role for Prenatal Nutritional Disruption in Metabolic Programming via Serotonergic Pathways						
Population/Model	Nutritional Exposure	Tissue Examined	Generation	Primary Outcome	Key Finding	Major Limitation
**Association Between Prenatal Famine Exposure and Later Metabolic Outcomes**						
Dutch Hunger Winter (Ravelli 1976; Ravelli 1999; Roseboom 2001) [36,37,38]	Acute wartime famine, November 1944–May 1945; trimester-specific timing well documented	Clinical/anthropometric	F1	Obesity (BMI); T2DM; metabolic syndrome	Exposure during first half of pregnancy associated with significantly elevated obesity rates at military induction (age 18–20); dose response with gestational timing; third trimester exposure showed non-significant reduction; later follow-up showed T2DM and metabolic syndrome	Single Western cohort; wartime confounders (stress, infection); no tryptophan or serotonin measurements
Chinese famine 1959–1961 (Li Y 2010; Liu 2019; Huang 2010; Li C 2024) [39,40,41,44]	Nationwide nutritional deficiency; severity varies by province and season; multiple concurrent deficiencies	Clinical/anthropometric	F1	Hyperglycemia; T2DM; abdominal obesity	Prenatal exposure associated with significantly elevated T2DM and obesity risk in large cohorts; sex-differentiated and trimester specific effects	Retrospective design; caloric vs. nutrient specific intake unknown; multiple concurrent deficiencies; political famine confounders
Holodomor, Ukraine 1932–1933 (Lumey 2024) [43]	Politically induced famine; severe caloric restriction; mortality approximately 4 million	Clinical registry	F1	T2DM incidence (adult)	Fetal exposure associated with adult T2DM; effect persists after adjustment for childhood conditions	Retrospective; exposure quantification limited; tryptophan pathway unmeasured
Great Hunger, Ireland 1845–1852 (Walsh 2012; indirect evidence) [29]	Sustained macronutrient restriction; maize substitution diverted tryptophan to niacin synthesis	Psychiatric registry (indirect)	F1	Elevated psychiatric admissions 1860–1875	Walsh documented elevated psychiatric admissions coinciding with birth cohorts gestated during the Hunger; consistent with serotonergic developmental disruption hypothesis	No birth cohort data; psychiatric outcome only; causal attribution speculative; multiple alternative explanations equally plausible
Finnish famines (Doblhammer 2013; Young 2017; Hayward 2013) [45,46,47]	Localized 19th century famines; severity and nutritional composition variable	Church records/registry	F1	Mortality; reproductive success; life expectancy	Early life nutritional adversity associated with reduced longevity and reproductive fitness; supports broader developmental-origins framework	Outcomes are life history, not metabolic; nutritional specifics uncharacterized; serotonin pathway not examined; effects small
Palestinian food insecurity (Mikki 2009; El Kishawi 2016; Al-Lahham 2019) [48,49,50]	Chronic household food insecurity; not a discrete historical famine; ongoing, multigenerational	Cross-sectional survey	Contemporary F0	Overweight/obesity prevalence across demographic groups	Consistent co-occurrence of food insecurity and elevated overweight/obesity in Gaza and West Bank; pattern consistent with developmental-programming prediction	Cross-sectional only; no prenatal exposure data; no tryptophan, serotonin, or epigenetic measurements; causality cannot be established
**Persistent epigenetic differences following prenatal famine exposure**						
Dutch Hunger Winter (Heijmans 2008) [30]	Periconceptional famine exposure; November 1944–May 1945	Whole blood (leukocytes)	F1, approximately 60 years post exposure	DNA methylation at IGF2 differentially methylated region (DMR)	Persistent hypomethylation at IGF2 DMR in periconceptionally exposed individuals (0.488 vs. 0.515; *p* = 0.000059); effect persists for more than six decades; present irrespective of sex or current metabolic status; specific to periconceptional exposure window	Peripheral blood proxy for hypothalamic tissue; effect size modest; serotonin pathway not measured; mechanism of persistence unresolved
Dutch Hunger Winter (Tobi 2014) [31]	Famine exposure across gestational trimesters	Whole blood (leukocytes)	F1	Genome-wide DNA methylation (Illumina 450K array)	Multiple CpG loci differentially methylated; enriched in metabolic and growth-regulatory gene regions; trimester-specific patterns identified	Tissue specificity limited to peripheral blood; replication cohort small; functional consequences of individual loci unclear
Chinese famine/Suihua cohort (Jiang 2020) [35]	Prenatal famine 1959–1961; Suihua county cohort	Whole blood	F1 and F2	Genome-wide DNA methylation (blood); eGFR association; autophagy regulatory loci	Blood DNA methylome analysis (138 subjects, two generations) identified 961 DMSs in F1 and 503 DMSs in F2; 19 sites conserved across both generations at autophagy-regulatory loci (CUX1, PPARGC1A, ELMO1, AGTR1); consistent with starvation-induced epigenetic memory of energy metabolism	Single county cohort; tissue specificity not addressed; serotonin pathway not examined
**Altered serotonin-related methylation in peripheral blood**						
Chinese famine/Suihua cohort (Zeng 2023) [12]	Prenatal caloric restriction 1959–1961; Suihua, Heilongjiang province	Whole blood (DNA methylation array)	F1	Methylation of 17 serotonin receptor signaling pathway genes	Reduced methylation at multiple 5-HTR loci in famine exposed individuals; effect predominantly in females; 17 genes in serotonin receptor signaling pathway affected	Whole blood is not hypothalamic tissue; reduced methylation does not necessarily indicate reduced expression; female predominant findings require sex-specific replication; causal link to obesity unconfirmed
**Changes in hypothalamic receptor expression in animal models**						
Rat model—Sprague-Dawley (Martin-Gronert 2016) [10]	Maternal protein restriction: 20% to 8% casein throughout pregnancy; not tryptophan-specific	Hypothalamus (ARC dissection)	F1, male offspring only	5-HT2A and 5-HT2C receptor mRNA and protein expression; food intake; body weight	Maternal protein restriction reduced hypothalamic 5-HT2A and 5-HT2C expression on POMC neurons; F1 males showed hyperphagia and late onset obesity on normal chow	Male rats only; sex specificity unresolved; general protein restriction rather than tryptophan specific manipulation; cross-species generalization uncertain; not replicated in human hypothalamic tissue
**True germline (transgenerational) transmission**						
No human cohort evidence (Eichenauer & Ehlert 2021 Dutch/Chinese famine cohorts) [32]	Multigenerational follow-up of Dutch and Chinese famine cohorts	Clinical/anthropometric (F2)	F2	Metabolic phenotype in grandchildren of famine exposed individuals	Some F2 metabolic differences reported; consistent with but not confirmatory of germline transmission; effect sizes small and inconsistent across cohorts	F2 observations do not exclude shared environment, cultural transmission, or maternal physiological effects; germline epigenetic mark persistence in humans not demonstrated; no serotonin pathway data in F2
Animal models (rodent) (González-Rodríguez et al. 2019, protein-restriction lineages) [34]	Maternal protein restriction across multiple generations	Germ cells	F2-F3	Persistence of metabolic phenotype across non-exposed generations	Some rodent lineages show phenotypic transmission beyond F1; epigenetic marks at relevant loci persist in germ cells across generations	Mechanism incompletely characterized; human equivalent not established; whether serotonin receptor methylation specifically undergoes germline inheritance is untested

ARC = arcuate nucleus; BMI = body mass index; CpG = cytosine-phosphate-guanine dinucleotide; DMR = differentially methylated region; eGFR = estimated glomerular filtration rate; 5-HT = serotonin; 5-HTR = serotonin receptor; OR = odds ratio; POMC = pro-opiomelanocortin; T2DM = type 2 diabetes mellitus.

## Data Availability

No new data were created or analyzed in this study. Data sharing is not applicable to this article.
